# TNFR1 expression on cancer cells mediates immune escape and an immunosuppressive microenvironment

**DOI:** 10.1084/jem.20252080

**Published:** 2026-09-07

**Authors:** Elixabet Bolaños, Lorena Cañas-Zabala, Irene Olivera, David Ruiz Guillamón, Enric Vercher, Gabriel Gomis, Carlos E. de Andrea, Raluca Alexandru, Arantza Azpilikueta, Nekane Soria, Dayanna Salinas, Paula Molero-Glez, Carlos Luri-Rey, Saioa Arrieta-Aranzueque, Almudena Manzanal, Mario Garcia-Dominguez, Diego Alignani, Bruno Ségui, Angie Molina, Álvaro Teijeira, Pedro Berraondo, Ignacio Melero

**Affiliations:** 1 Program of Immunology and Immunotherapy, CIMA Universidad de Navarra, Cancer Center, Clínica Universidad de Navarra (CCUN), Pamplona, Spain; 2 Navarra Institute for Health Research (IDISNA), Pamplona, Spain; 3 Department of Pathology, Anatomy and Physiology, Clinica Universidad de Navarra, Cancer Center Clinica Universidad de Navarra (CCUN), University of Navarra, Pamplona, Spain; 4 Department of Immunology and Immunotherapy, Clinica University of Navarra, Cancer Center Clínica Universidad de Navarra (CCUN), Pamplona, Spain; 5 Centro de Investigación Biomédica en Red de Cáncer (CIBERONC), Madrid, Spain; 6 Unité Mixte de Recherche INSERM 1037, CNRS 5071, Université Toulouse III-Paul Sabatier, Centre de Recherches en Cancérologie de Toulouse (CRCT), Toulouse, France; 7 Nuffield Department of Medicine (NDM), University of Oxford, Oxford, UK

## Abstract

TNFα is a proinflammatory cytokine that can mediate immunosuppressive effects in cancer. Tumors in which we silenced TNFR1 by CRISPR/Cas9 showed that TNFR1KO variants poorly engrafted in immunocompetent hosts, while engraftment in immunodeficient or CD8 T lymphocyte–depleted mice was preserved. The mechanism was mediated by secondary chemotactic inflammatory mediators elicited by TNFα in the malignant cells themselves. As a result, TNFR1KO variants recruited drastically fewer myeloid-derived suppressor cells (MDSCs) into the tumor microenvironment, thus explaining the immune escape of the WT variants. Interestingly, small amounts of TNFR1-sufficient tumor cells co-engrafted with the TNFR1KO variants rescued tumorigenicity in the same tumor lesion but not in distantly implanted TNFR1KO tumors. Secondary mediators chiefly include CXCR1/2-acting chemokines, prostaglandin E_2_ and TNFα itself. Knocking down TNFR1 in a human tumor cell line rendered comparable MDSC-recruiting results when xenografted. Analyses of scRNA-seq and spatial transcriptomics datasets from human solid tumors corroborate the role of TNFR1 on tumor cells in the induction of secondary pro-tumor inflammatory mediators.

## Teaser

This study provides evidence for a key role of TNFR1, as expressed by malignant cells, in orchestrating a pro-tumor immunosuppressive environment through secondary inflammatory mediators. Results highlight the relevance of interfering TNFα/TNFR1 in cancer immunotherapy.

## Graphical Abstract

**Figure:**
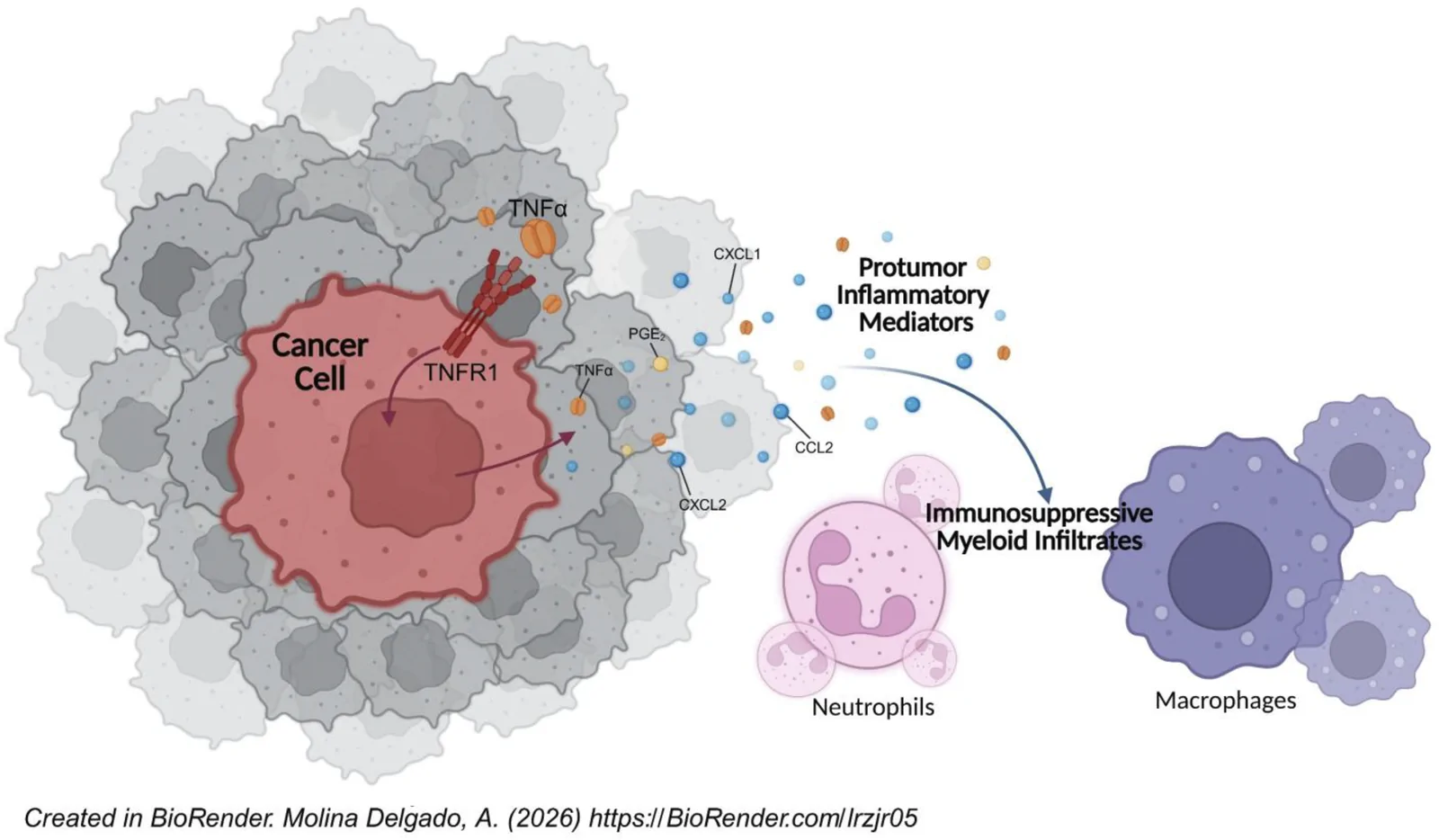

## Introduction

Tumor necrosis factor α (TNFα) owes its name to the original observation that intratumoral injection of this cytokine was able to induce necrosis of engrafted tumors in rats (Carswell et al., 1975). This antitumor effect was mainly mediated by vascular damage rather than by direct induction of tumor cell apoptosis (Robaye et al., 1991; Stoelcker et al., 2000). Despite the fact that most tumor cells do express TNFR1, it is often uncoupled from the apoptosis-inducing signalling machinery (Spriggs et al., 1987; Chen et al., 2021; Wang and Lin, 2008; Brenner et al., 2015). The antitumor effects of TNFα were exploited to develop isolated limb perfusion strategies, which demonstrated clinical efficacy (Eggermont et al., 1996; Grünhagen et al., 2006). The TNFα gene was also cloned under the name cachectin because of its ability to induce cachexia in mice (Tracey and Cerami, 1990). Indeed, TNFα is one of the cytokines involved in cachexia pathogenesis in advanced cancer patients (Matthys and Billiau, 1997).

The group of F. Balkwill pioneered experimental evidence showing that TNFα functions primarily as a pro-tumor rather than an antitumor cytokine (Naylor et al., 1993; Balkwill et al., 1987; Balkwill, 2009; Balkwill and Joffroy, 2010). Indeed, TNFα neutralization exerted therapeutic effects in cancer animal models (Scott et al., 2003). Such work was conducive to two clinical trials in cancer patients blocking TNFα with infliximab used as a monotherapy, which resulted in modest antitumor activity (Madhusudan et al., 2004; Madhusudan et al., 2005). The current consensus is that the pro-tumor rather than the antitumor effects of TNFα predominate in cancer patients (Alim et al., 2024).

Our group demonstrated in tumor-bearing mice treated with checkpoint-inhibitor immunotherapy that TNFα neutralization led to substantially enhanced efficacy of anti–PD-1 plus anti–CTLA-4 immunotherapy, while also mitigating immune-mediated side effects (Perez-Ruiz et al., 2019). Similar observations were made under PD-1 single blockade (Bertrand et al., 2017). These studies provided evidence of the cancer-associated immunosuppressive activities of TNFα. Given these results, a phase Ib trial was conducted in small cohorts of advanced melanoma patients, offering promising results for TNFα neutralization with certolizumab in combination with double nivolumab plus ipilimumab checkpoint blockade both in terms of efficacy and safety (Montfort et al., 2021).

TNFα is not the only proinflammatory factor that orchestrates immunosuppression in cancer. Indeed, there is evidence that IL-6, IL-1α/β, CXCR1/2-acting chemokines, LIF, CSF-1, or VEGFs cause pro-tumor immunosuppressive inflammation (Olivera et al., 2023; Propper and Balkwill, 2022). Therefore, neutralization of these inflammatory mediators has been reported to also synergize with checkpoint inhibitors in cancer mouse models (Perez-Ruiz et al., 2019; Mantovani et al., 2018; Ries et al., 2014; Hallett et al., 2023; Hailemichael et al., 2022; Bertrand et al., 2017).

A number of mechanisms that mediate TNFα immunosuppression in cancer models have been shown. These include activation-induced cell death of antitumor T lymphocytes (Perez-Ruiz et al., 2019; Otano et al., 2020), dendritic cell dysfunction (Alam et al., 2024), and the elicitation of chemokines that attract myeloid-derived suppressor cells (MDSCs) (Olivera et al., 2022). Other yet-to-be-reported mechanisms might also be at play for pro-tumor TNFα activities.

In this study, we show that malignant-cell sensitivity to TNFα via TNFR1 is critical to avoid immune rejection of a variety of transplantable tumors and genetically induced hepatocellular carcinoma (HCC). Interestingly, TNFα induces the expression of several secondary inflammatory mediators in tumor cells, which ultimately shape an immunosuppressive tumor tissue microenvironment.

## Results and discussion

### TNFR1 loss in tumor cell lines results in immune-mediated spontaneous regression

**Figure S1.**
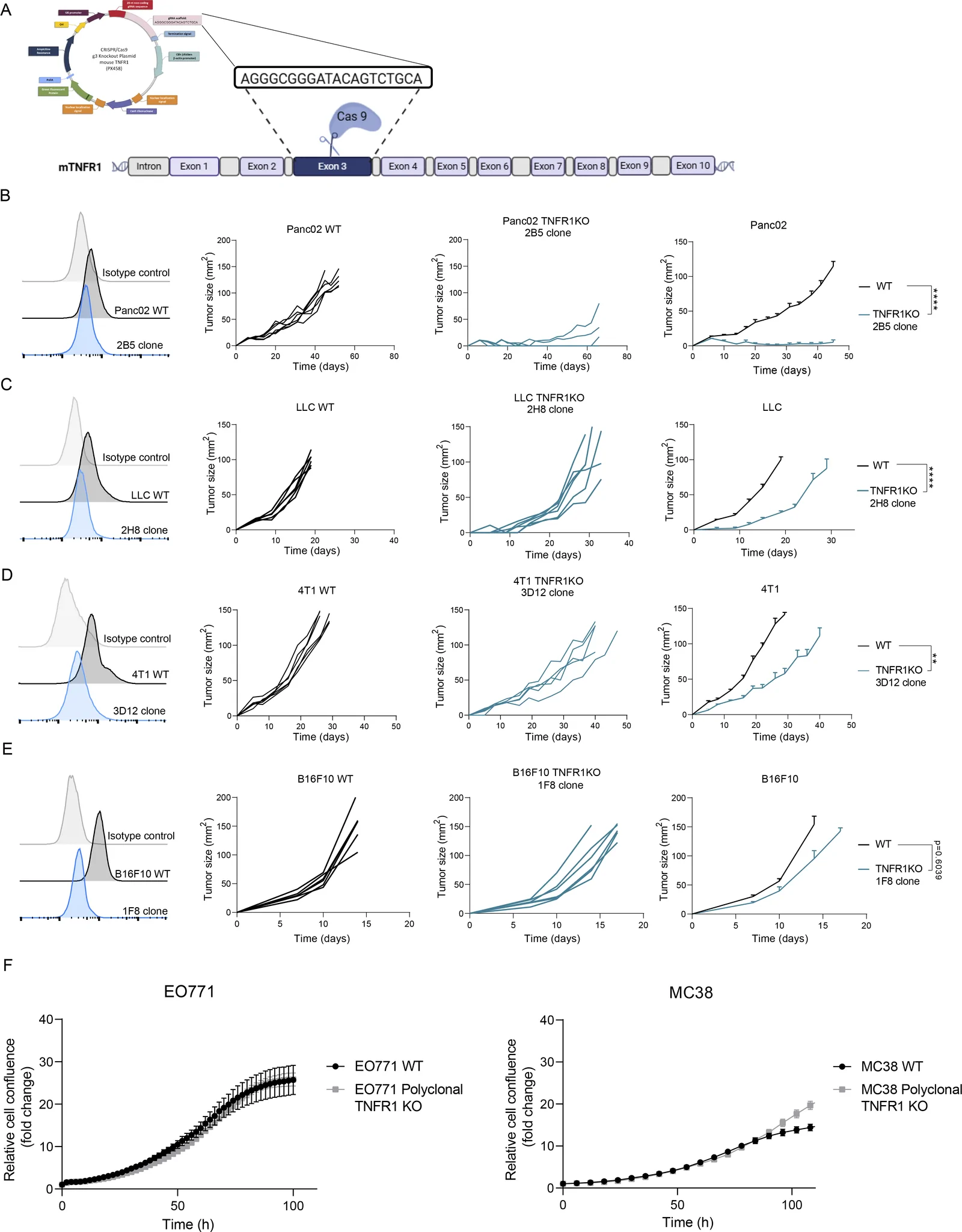
**Knocking down TNFR1 in transplantable murine tumor cell lines. (A)** CRISPR/Cas9 expression plasmid used to silence TNFR1 expression. The sequence in the dotted square corresponds to the selected TNFR1 gRNA. **(B–E)** Similar experiments as in Fig. 1, A–D with TNFR1 WT and KO variants derived from the Panc02, LLC, 4T1, and B16F10 tumor cell lines (two-way ANOVA statistical comparisons) (*n* = 6). **(F)** Incucyte assessment of proliferation of the indicated WT or TNFR1KO variants of EO771 (left) and MC38 (right). P < 0.01 (**) and P < 0.0001 (****).

**Figure 1.**
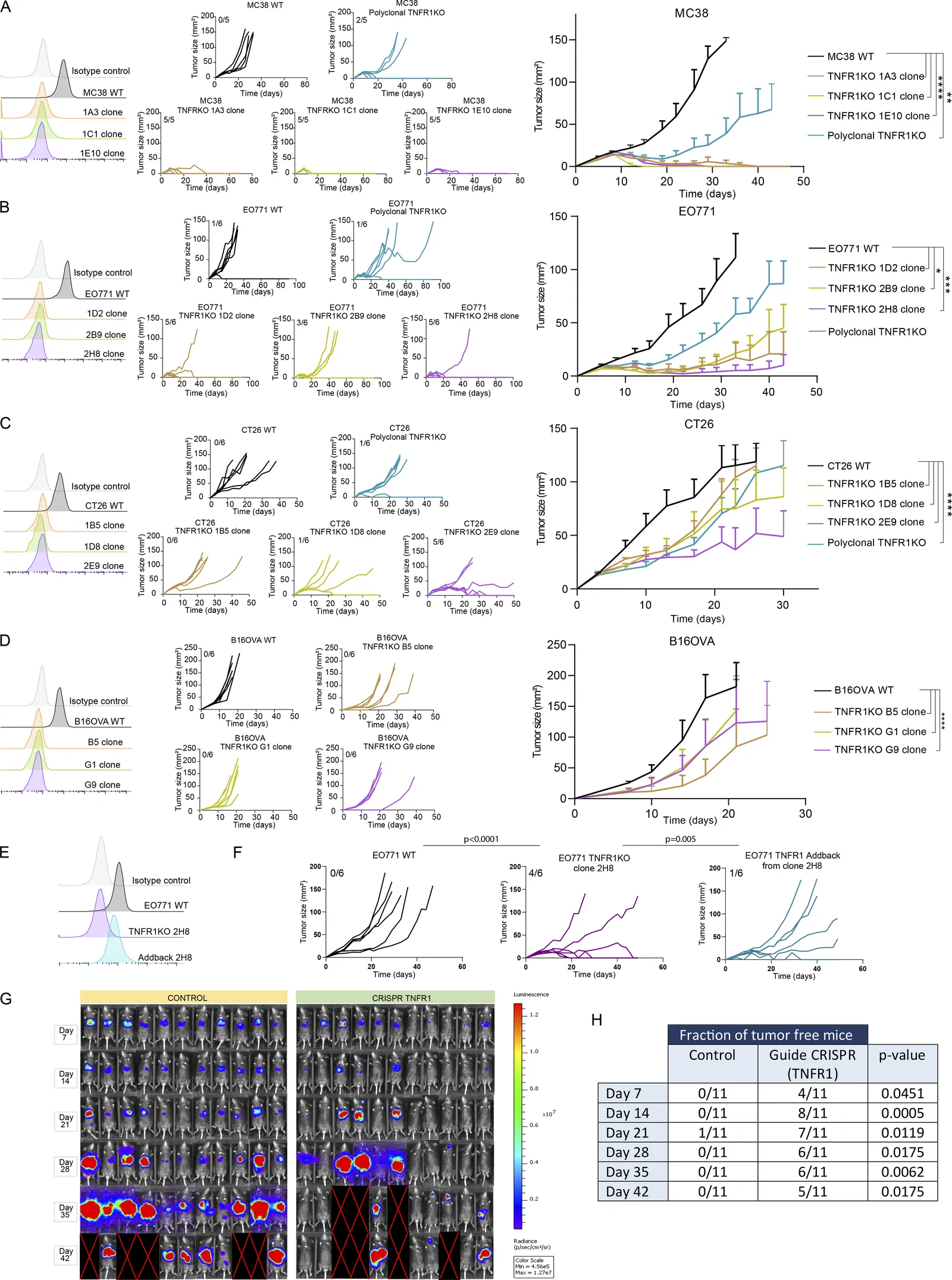
**TNFR1KO variants are rejected or slowly progress in transplantable models and in a genetically induced multifocal HCC model. (A–D)** TNFR1KO clonal variants of the indicated transplantable cell lines were subcutaneously engrafted in immunocompetent syngeneic mice in comparison to their WT cell counterparts. Flow cytometry histograms showing the expression or silencing of TNFR1 (on the left). Individual tumor follow-up and lethal tumor engraftment (left panels) are presented alongside summaries of data (mean ± SEM in the right panels, also providing two-way ANOVA statistical comparisons). Fractions in the top left corner of each graph show the number of mice that experienced complete tumor rejection (*n* = 5–6). **(E)** FACS histograms showing TNFR1 expression in EO771 WT, EO771 TNFR1KO (clone 2H8), and its lentivirally transduced back with TNFR1 to regain expression. **(F)** Tumor engraftment experiments in immunocompetent syngeneic mice of the WT and TNFR1KO variant of EO771 in comparison with the KO variant lentivirally transduced to regain expression of TNFR1 (unpaired *t* test statistical comparisons) (*n* = 6). **(G)** Tumor generation and individual follow-up by sequential bioluminescence imaging of multifocal HCC induced by hydrodynamic gene co-transfer of plasmids to express cMyc, GFP, and luciferase with a CRISPR/cas9 silencing system to knock down p53. As indicated in the figure, in a group of mice, the TNFR1 CRISPR guide was added to the hydrodynamic mixture and controlled by an empty vector in the other group. **(H)** Fraction of mice without evidence of tumors at the indicated time points and Fisher’s exact test statistical comparisons (*n* = 11). Experiments are representative of at least two similarly performed. P < 0.05 (*), P < 0.01 (**), P < 0.001 (***), and P < 0.0001 (****).

In previous work, we reported that TNFα and IL-1β were able to stimulate the production of CXCR1/2 chemokines from tumor cells, an effect that was prominent in the case of TNFα acting on TNFR1 (Olivera et al., 2022). To study the relevance of such mechanisms, we used CRISPR/Cas9-mediated deletion (Fig. S1 A) of the TNFR1 gene in transplantable mouse tumor cell lines with various degrees of intrinsic immunogenicity, including MC38 (colon cancer), EO771 (breast cancer), CT26 (colon cancer), and B16OVA (melanoma). As shown in Fig. 1, A–D, we generated multiple stably silenced clones and created mixtures of polyclonal TNFR1 KO populations to avoid artefacts due to clonal heterogeneity (Keenan et al., 2026; Wolf et al., 2019) in subsequent experimentation.

We subcutaneously injected the TNFR1KO clonal variants in comparison with the WT counterparts in groups of syngeneic mice. Fig. 1, A–D, shows that the TNFR1KO clonal variants were often rejected after transient or delayed growth in the case of MC38, EO771, and CT26, while in poorly immunogenic B16OVA tumors, a longer tumor latency and slower progression were observed for the TNFR1KO clones. Of note, differences in tumor intake and progression cannot be explained by the different rate of cell division in culture (Fig. S1 F).

Similar effects were generalized to Panc02 pancreatic cancer, Lewis lung carcinoma, 4T1 breast cancer, and, to some extent, B16F10 melanoma (Fig. S1, B–D). To further confirm the role of TNFR1, we reintroduced *Tnfr1* by lentiviral transduction into an EO771-silenced variant (Fig. 1 E). Experiments in Fig. 1 F show a clear recovery of tumor engraftment.

To study whether such phenomena could be observed in autochthonous genetically induced tumors in mice, we took advantage of the hydrodynamic gene-transfer model of HCC (Ochoa et al., 2023; Ruiz de Galarreta et al., 2019). In this model, co-injection of expression plasmids encoding p53 silencing with CRISPR/Cas9 and cMyc results in rapidly progressing multifocal HCC, which can be traced with bioluminescence imaging because of the luciferase gene co-transfer. In this genetic model, we tested the hydrodynamic co-injection of the plasmid encoding the guide to knock down TNFR1. As shown in Fig. 1 G, silencing TNFR1 resulted in rejection or slower progression of the multifocal liver tumors, as statistically compared with those that did not receive the TNFR1 guide upon oncogenic hydrodynamic gene co-transfer (Fig. 1 H).

TNFR1 is a receptor that encompasses a death domain in its intracellular sequence (Tartaglia et al., 1993; Hsu et al., 1995) but which, in our hands, does not induce tumor cell death upon TNFα exposure. Cancer cells resist TNFα-induced apoptosis through mechanisms such as the activation of anti-apoptotic molecules (e.g., IAPs, Bcl-2), via the NF-κB signalling pathway, or through overexpression of intracellular inhibitors like cFLIP (Montfort et al., 2019). It is curious that despite its potential proapoptotic functions and the availability of TNFα in the tumor microenvironment, expression of TNFR1 is preserved in the majority of cancer cell lines, indicating a predominant pro-tumor activity.

The relevance of TNFR1 seems to be greater in tumors with a higher degree of immunogenicity/antigenicity. Our observations are not limited to transplantable models but were also substantiated in a genetic multifocal HCC model that has been used to study combined immunotherapies (Ochoa et al., 2023). TNFR1 has also been reported to play a role in an immunogenic mouse melanoma model, when expressed on cells from the tumor microenvironment, including CD8^+^ TILs (Bertrand et al., 2015). In fact, the tumor growth of B16K1 melanoma cells, which overexpress MHC-I, was significantly impaired in TNFR1KO mice. Accordingly, our results do not disregard other reported immunosuppressive effects of TNFα on other cells of tumor-bearing hosts, but highlight a dominant role orchestrated by tumor cells themselves when sensing TNFα. Therefore, direct deleterious TNFα effects on T cells via activation-induced cell death (Perez-Ruiz et al., 2019; Otano et al., 2020) or dendritic cell inhibition (Alam et al., 2024) coexist and probably act in collusion as a result of secondary proinflammatory factors that we hypothesized to be produced by malignant cells under TNFα influence (Olivera et al., 2022).

### TNFR1KO variants are rejected through immune-mediated mechanisms

To study whether the tumor rejections were mediated by the immune system, we performed experiments in T and B cell–deficient Rag1^−/−^ mice and selective depletions of CD4 and CD8_β_ T cells with monoclonal antibodies.

**Figure 2.**
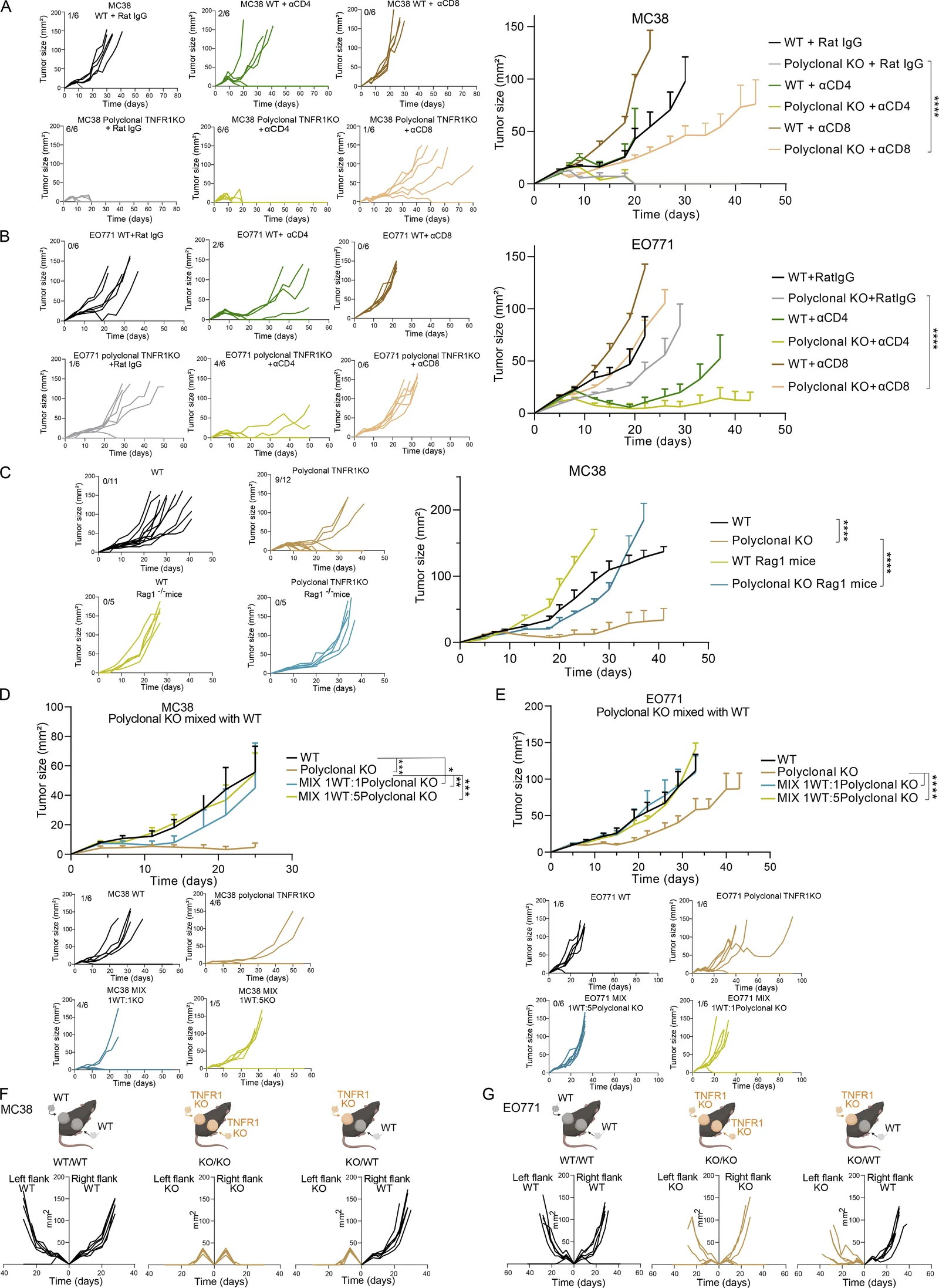
**TNFR1KO tumor variants are controlled by CD8** ^**+**^ **T lymphocytes, and small quantities of TNFR1** ^**+**^ **co-injected variants locally rescue tumorigenicity of TNFR1KO tumor cells. (A)** Selective T cell depletion experiments in mice bearing WT or TNFR1KO MC38 cells treated with control antibody or depleted of CD4^+^ or CD8_β_^+^ lymphocytes, with the lower graph presenting cumulative data (mean ± SEM) and two-way ANOVA statistical comparisons (*n* = 6). **(B)** Results as in A but with EO771 WT and TNFR1KO variants. Fractions in the top left corner of individual tumor graphs show the number of mice that experienced tumor rejection (*n* = 6). **(C)** Individual follow-up of tumor sizes in WT or Rag1^−/−^ mice engrafted subcutaneously with the indicated WT or TNFR1KO MC38 variants. The graph on the right summarizes data (mean + SEM) and provides two-way ANOVA statistical comparisons (*n* = 5–12). **(D and E)** Experiments as in Fig. 1 but inoculating either 1:1 or 1:5 mixtures of the WT and TNFR1KO variants, using MC38 (D) or EO771 (E) cells, respectively. Cumulative data (mean ± SEM) and two-way ANOVA statistical comparisons are presented in the figures (*n* = 6). **(F and G)** Experiments testing subcutaneous contralateral inoculation of tumor cells, either WT (represented in black) or TNFR1KO (represented in light brown). Individual follow-up of right and left subcutaneous tumors is shown. F refers to MC38 variants, while G corresponds to EO771 variants. The shown experiments are representative of two replicates, with the exception of F and G, which represent a single experiment. P < 0.05 (*), P < 0.01 (**), P < 0.001 (***), and P < 0.0001 (****).

To avoid clonal heterogeneity, we generated polyclonal TNFR1KO variants by mixing at least three clones in equal proportions while in culture immediately before engrafting. Using polyclonal TNFR1KO MC38, we confirmed rejection of all the tumors in immunocompetent mice (Fig. 2 A). CD4 depletion did not alter rejection, while CD8_β_ T cell depletion completely abrogated rejection, even if the tumors still progressed less aggressively than the WT counterparts (Fig. 2 A). In the case of EO771, the clear delay in the progression of the TNFR1KO polyclonal variants was also abrogated by CD8_β_ depletion, whereas the elimination of CD4 T cells reduced tumor growth in both WT and TNFR1KO EO771 cells, possibly due to regulatory T cell depletion (Fig. 2 B).

**Figure S2.**
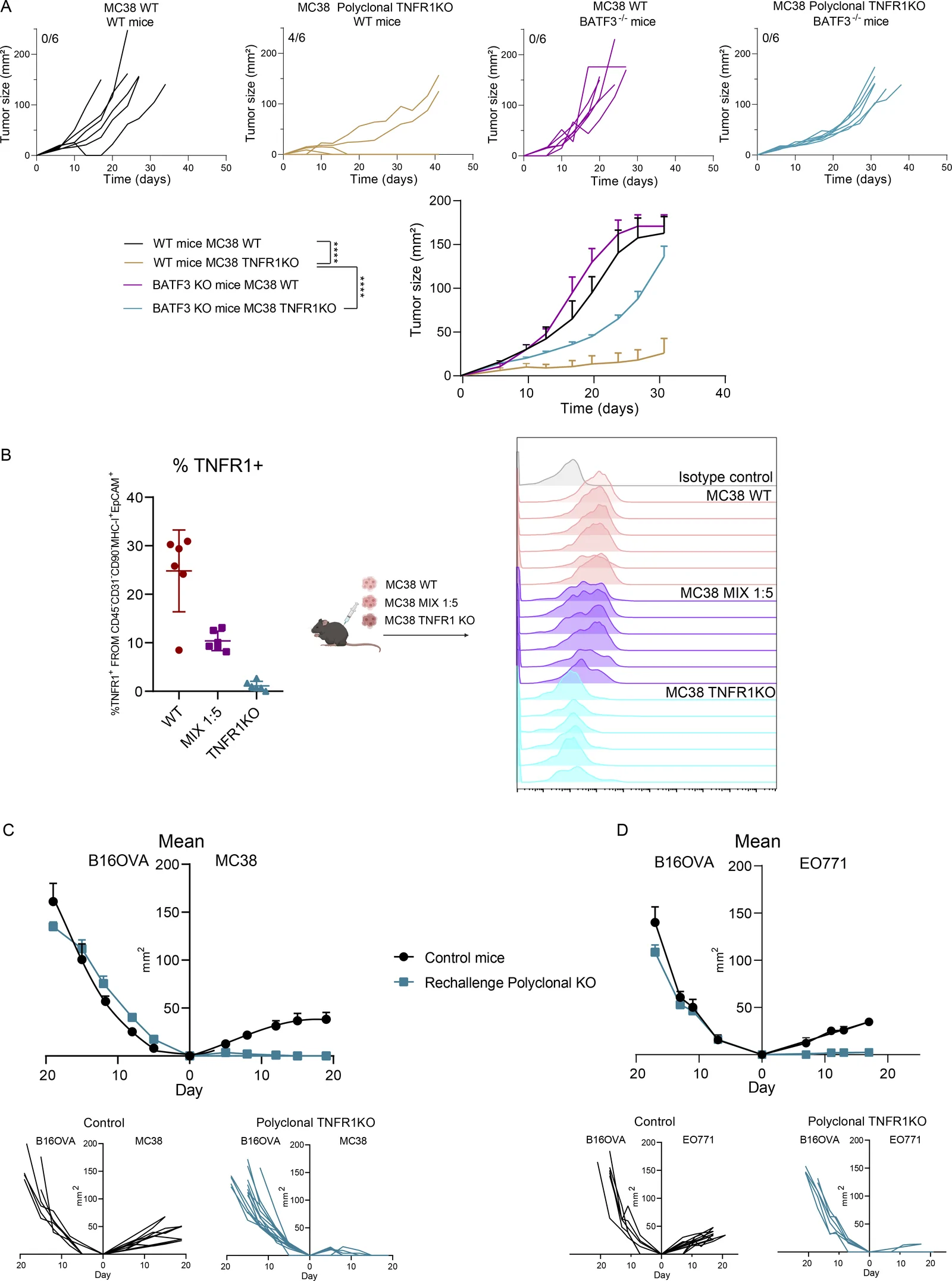
**TNFR1KO variants regain tumorigenicity in cDC1-deficient BATF3** ^**−/−**^ **mice, in vivo assessment of TNFR1 WT:KO mixtures in Fig. 2, D and E, and tumor rechallenge experiments. (A)** Comparative tumor engraftment of the indicated MC38 cell variants in WT and BATF3^−/−^ mice is shown by individual follow-ups and mean ± SEM with two-way ANOVA statistical comparisons (lower graph) (*n* = 6). **(B)** FACS assessment of TNFR1 immunostaining of cell suspensions from tumors in Fig. 2 D, including the indicated mixtures. Each dot represents data from an individual tumor (*n* = 6). On the right, individual histograms are shown. **(C and D)** Rechallenge of the mice rejecting TNFR1KO tumors in experiments as in Fig. 1, A and B, that were rechallenged >90 days later with MC38 WT (C) or EO771 WT (D) and antigenically unrelated B16OVA in the contralateral flank (*n* = 6–12). Upper graphs in C and D represent mean ± SEM, and individual tumor follow-up is provided in the lower graphs. P < 0.0001 (****).

The involvement of immune surveillance was further confirmed in experiments performed comparing tumor intake and growth in WT, Rag1^−/−^ (Fig. 2 C), as well as in BATF3^−/−^ mice (Fig. S2 A). Indeed, TNFR1KO MC38 polyclonal variants grew aggressively in Rag1^−/−^ mice, while they were rejected in WT syngeneic mice (Fig. 2 C). BATF3^−/−^ mice are deficient in cDC1 dendritic cells that are necessary to mount CD8 cytotoxic T cell responses (Hildner et al., 2008). As can be seen in Fig. S2 A, TNFR1KO MC38 cells aggressively engrafted and grew in BATF3^−/−^ mice. Together, these data demonstrate that rejection of TNFR1KO variants is mediated by CD8 immune responses, although we cannot completely exclude a role for CD4 T cells or other lymphocyte subsets explaining the differences between CD8 depletion in WT mice and observations in Rag1^−/−^ mice.

### Local co-injection of TNFR1-expressing tumor cells rescues tumorigenicity in immunocompetent mice

To start addressing potential mechanisms behind the increased immunogenicity of TNFR1KO tumor cell variants, we co-injected different proportions of TNFR1-sufficient and deficient cells. Fig. 2, D and E show that as few as one in five TNFR1-sufficient cells rescued tumorigenicity in immunocompetent mice. Indeed, this proportion was preserved in in vivo transplanted tumors over 8 days (Fig. S2 B).

We reasoned that in response to TNFα, TNFR1^+^ tumor cells produced some factor(s) that would rescue the engraftment of TNFR1-silenced variants in trans, modifying the immune microenvironment of the tumor as a whole and thereby rescuing neighboring TNFR1KO cells.

Consistent with this idea, we subcutaneously co-engrafted TNFR1KO and TNFR1 WT variants in distant flanks. In this setting, as shown in Fig. 2, F and G, the TNFR1KO variants experienced immune control while the concomitant TNFR1-sufficient tumors progressed in the same mouse. These results indicated that the rescue requires the TNFR1^+^ and TNFR1^−^ tumor cells to coexist in close proximity.

In mice that had rejected MC38 TNFR1KO variants, MC38 WT did not engraft in the contralateral flank, while unrelated B16OVA-derived tumors readily progressed (Fig. S2 C). Similar observations were made in mice that had rejected EO771 TNFR1KO variants (Fig. S2 D).

We were initially surprised that TNFR1 has not been identified as a gene conferring immune escape in many reported CRISPR/Cas9 screenings for immune resistance (Sun et al., 2023; Tsao et al., 2024; Wu et al., 2024). However, the fact that TNFR1-sufficient tumor cells can rescue neighboring TNFR1-negative variants likely explains the lack of disappearance or enrichment of such CRISPR guides in these genetic screenings. Indeed, the effect of secondary inflammatory mediators likely favors those tumor cells in the vicinity, as shown in our co-injection experiments. Nonetheless, this effect does not manifest when TNFR1^+^ and TNFR1^−^ tumors are implanted distantly from each other in the same mouse. Our experiments in Rag1^−/−^ mice and CD8 depletions indicate that pro-tumor activities of TNFα are more related to local immunosuppression than to intrinsic functions of the malignant cells.

### TNFα induces secondary inflammatory mediators in transplantable murine tumor cell lines that include TNFα itself and increases MDSC infiltration

**Figure 3.**
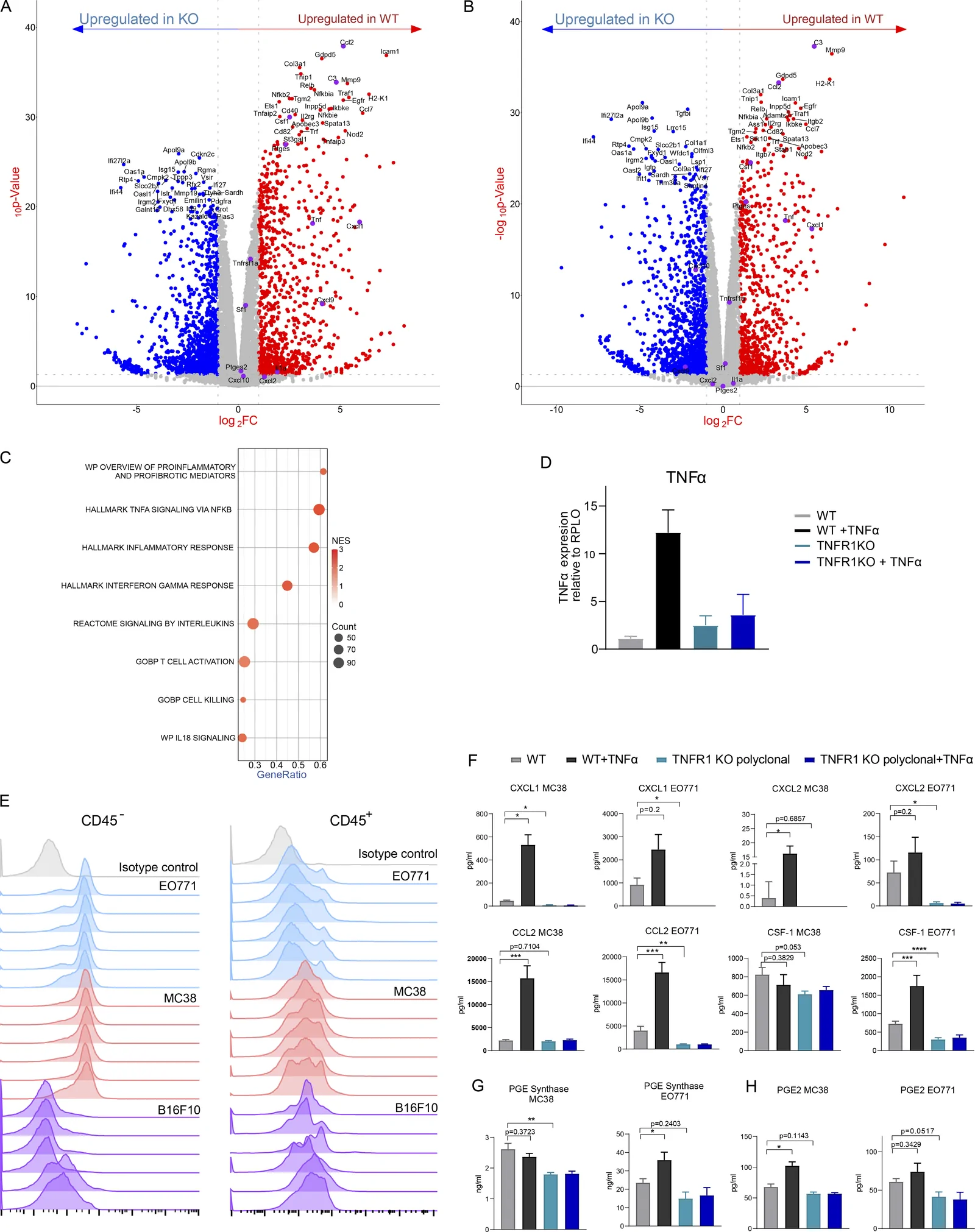
**TNFα induces the expression of multiple secondary inflammatory mediators in mouse tumor cells. (A and B)** Volcano plots showing genes induced or downregulated by TNFα in EO771 cells, as assessed by bulk RNA-seq 6 and 24 h following TNFα exposure of the cultures. The genes involved in pro-tumor inflammation are highlighted in purple. **(C)** Enrichment plot showcasing gene sets upregulated, including multiple proinflammatory pathways. **(D)** TNFα gene expression levels relative to RPL0 mRNA levels assessed by quantitative RT-PCR in WT and TNFR1KO variants. **(E)** Expression of membrane and intracellular TNFα in cell suspensions from the indicated excised tumors, gated on CD45^−^ (left histograms) and CD45^+^ cells (right histograms). **(F)** CXCL1, CXCL2, CCL2, and CSF-1 concentrations in the indicated 72 h cell culture supernatants of MC38 and EO771 cells after induction with rTNFα (*n* = 6). **(G)** Induction of PGE synthase protein assessed by ELISA in the same culture supernatants (*n* = 6). **(H)** PGE_2_ concentrations in supernatants measured by ELISA (*n* = 6). Bulk mRNA-seq was performed with three replicates, and experiments in D–F were analyzed by Mann–Whitney U test and performed at least twice with comparable results. P < 0.05 (*), P < 0.01 (**), P < 0.001 (***), and P < 0.0001 (****).

To understand the results of TNFR1KO variants, we decided to study the genes whose expression was changed upon the influence of rTNFα in the EO771 cell line. Bulk RNA sequencing (RNA-seq) at 6 and 24 h following TNFα exposure in culture induced a rich array of proinflammatory substances that are highlighted in purple in Fig. 3, A and B. Gene ontology (GO) analyses also highlighted these proinflammatory pathways (Fig. 3 C). It is important to note that the TNFα gene was induced in the tumor cells by TNFα, as confirmed by quantitative RT-PCR (Fig. 3 D), thereby potentially generating autocrine loops. An important question is what the cellular sources of TNFα are in the engrafted tumors. To address this point, we intracellularly stained for TNFα expression in tumor cell suspensions of EO771, MC38, and B16F10. Fig. 3 E shows TNFα expression in both gated CD45^−^ and CD45^+^ cells. Therefore, our results show that TNFα is prominently expressed both in tumor cells and leukocyte stroma, but less so in the case of B16F10 melanoma, perhaps explaining its lower dependency on TNFR1 (Fig. S1 E). Our results highlight the relevance of tumor-intrinsic autocrine TNFα and are consistent with our published observation in tumor lymphoid and myeloid infiltrates (Olivera et al., 2022).

Given the already known immunosuppressive effects in cancer models of the chemokines CXCL1, CXCL2, CCL2, as well as CSF-1 (Olivera et al., 2023), we studied the concentrations of such mediators in the cell culture supernatants of TNFR1^+^ and TNFR1^−^ variants of MC38 and EO771. As shown in Fig. 3 F, prominent increases at the protein level were only observed in rTNFα-treated WT cells, but not in the TNFR1KO variants.

The induction of prostaglandin E (PGE) synthase also caught our attention due to the published evidence of prostaglandin E_2_ (PGE_2_) involvement in tumor escape from the immune system (Bonavita et al., 2020). Indeed, we confirmed the increased expression of PGE synthase, resulting in higher concentrations of PGE_2_ measured in the culture supernatants (Fig. 3, G and H).

**Figure 4.**
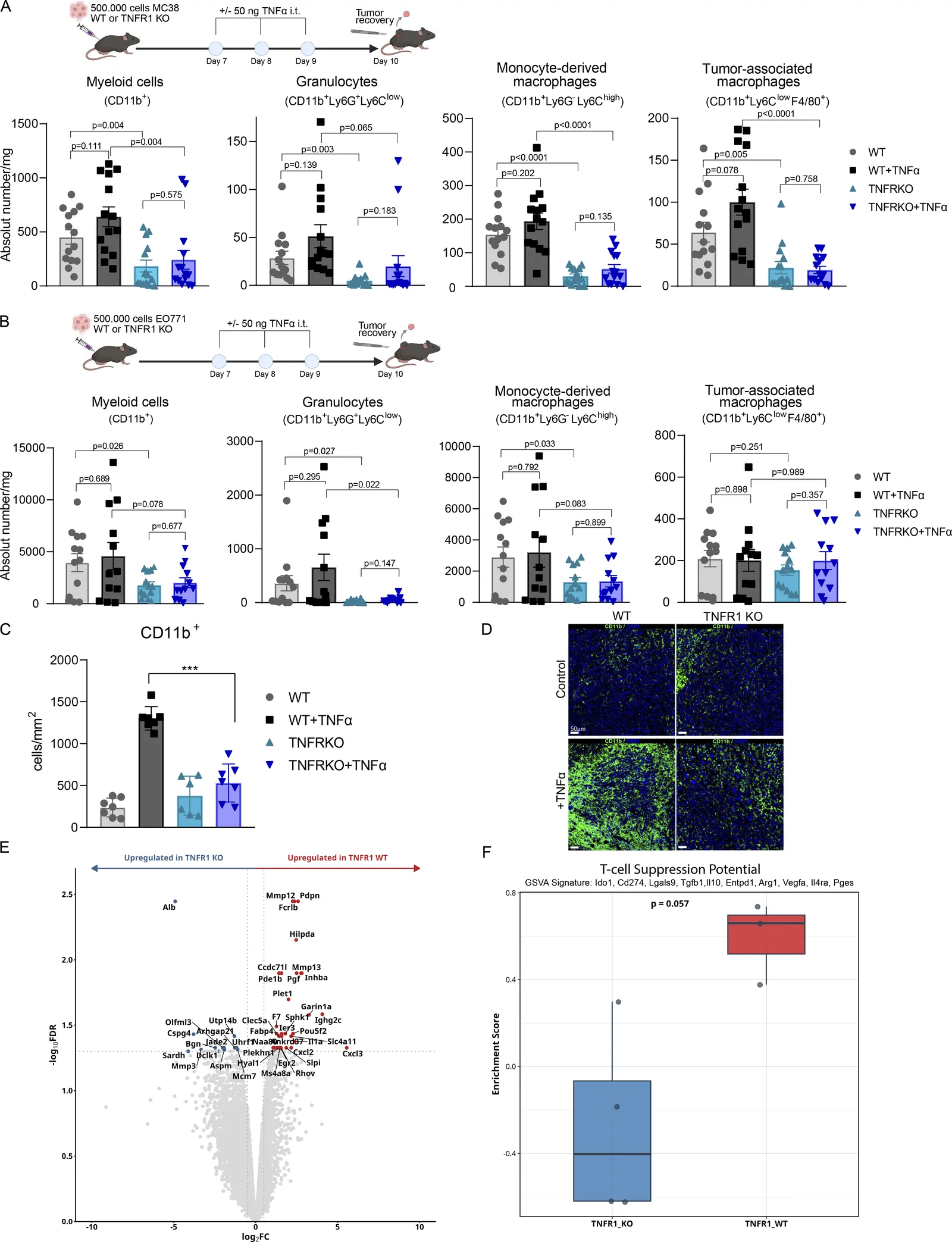
**TNFR1KO cells recruit fewer MDSCs into the tumor tissue microenvironment. (A)** Schematic representation of the experimental workflow in mice bearing tumors derived from either MC38 or MC38 TNFR1KO variants. Tumors were intratumorally injected with rTNFα as indicated and excised on day 10. Cell suspensions derived from such tumors were analyzed by multicolor flow cytometry to quantify the density of neutrophils (Gr-MDSC) and macrophages (M-MDSC). Each dot represents data from an individual tumor (*n* = 12–14). A *t* test was performed for statistical comparisons. **(B)** Experiments as in A with the EO771 variants (*n* = 12–14). A *t* test was performed for statistical comparisons. **(C)** Analysis by multiplex tissue immunofluorescence of the density of CD11b^+^ myeloid cells in the tumors, either injected or not with TNFα (*n* = 6–8). Statistical comparisons were performed by the Mann–Whitney U test. **(D)** Representative microscopy images from C (Scale bar: 50 µm). **(E)** CD11b^+^ tumor-infiltrating myeloid cells from TNFR1KO or WT EO771 tumors that were FACS-sorted and mRNA sequenced in triplicate. The volcano plot represents the main differentially expressed genes. **(F)** Comparison of myeloid suppressive signature (*Ido1*, *Cd274*, *Lgals9*, *Tgfb1*, *Il10*, *Entpd1*, *Arg1*, *Vegfa*, *Il4ra*, and *Pges*) in the mRNA-seq datasets from the sorted CD11b^+^ cells in E. Differential statistics were evaluated by the Mann−Whitney U test. P < 0.001 (***).

A conceivable potential mechanism was that secondary inflammatory mediators attract and activate immature neutrophils and monocytes, which then act as MDSCs (Bronte et al., 2016; Veglia et al., 2021). To ascertain such a phenomenon, we engrafted TNFR1KO and WT variants of MC38 (Fig. 4 A) or EO771 (Fig. 4 B) and injected TNFα intratumorally as indicated in Fig. 4, A and B. In recovered excised tumors, an increased number of Gr-MDSC (CD11b^+^Ly6G^+^Ly6C^low^) and M-MDSC (CD11b^+^Ly6G^−^Ly6C^high^) was observed upon TNFα injection, which was lost for the most part in the TNFR1KO variants as assessed by flow cytometry in tumor-derived cell suspensions (Fig. 4, A and B).

Tissue immunofluorescence results confirmed the evidence for a denser CD11b^+^ myeloid cell infiltration upon TNFα intratumoral injection that was lost when the malignant cells were TNFR1KO (Fig. 4, C and D). These findings indicate that the MDSC phenotype in response to TNFα injection is mainly mediated through tumor cells themselves rather than through stromal cells in the tumor microenvironment that would normally express TNFR1 in this experimental setup.

We favor CXCR1/2-acting chemokines and PGE_2_ as key factors, but further investigations should determine which ones are the most relevant, whether there is redundancy of effects, and which opportunities for therapeutic intervention these scenarios may offer. The immunosuppressive role of PGE_2_ in the tumor is well documented and therapeutically actionable (Bonavita et al., 2020; Zelenay et al., 2015).

To compare CD11b^+^ cells sorted from EO771 TNFR1 WT or TNFR1KO tumors, we performed bulk RNA-seq that identified differences in important functional genes involved in metalloproteases and chemokines (Fig. 4 E). Moreover, CD11b^+^ leukocytes from TNFR1 WT showed evidence for a more immunosuppressive gene score (*Ido1*, *Cd274*, *Lgals9*, *Tgfb1*, *Il10*, *Entpd1*, *Arg1*, *Vegfa*, *Il4ra*, and *Pges*) (Fig. 4 F).

Our studies identified TNFα as a gene induced by TNFα in tumor cells, in addition to lymphoid and myeloid leukocytes, perhaps fuelling an autocrine self-perpetuating loop. In any case, our results in Fig. 1 E speak of no shortage of TNFα availability in the TME. Other authors have linked tumor cell expression of TNFα to resistance to TNFR1-mediated apoptosis (Wang and Lin, 2008).

Therefore, as a result of being TNFα-sensitive, tumor cells shape a tissue environment enriched in MDSCs (Akkari et al., 2024) of both granulocyte and monocyte types. This, by itself, explains better tumor engraftment, especially in tumors known to be antigenic/immunogenic, such as MC38. In turn, myeloid leukocytes interfere with CD8^+^ T cell activation (Gabrilovich, 2017) and may also functionally affect the BATF3^−/−^ dependent dendritic cells that mediate tumor-antigen cross-presentation/cross-priming (Luri-Rey et al., 2025).

### TNFR1 expression in malignant cells of human cancer is associated with the expression of secondary pro-tumor inflammatory mediators

**Figure 5.**
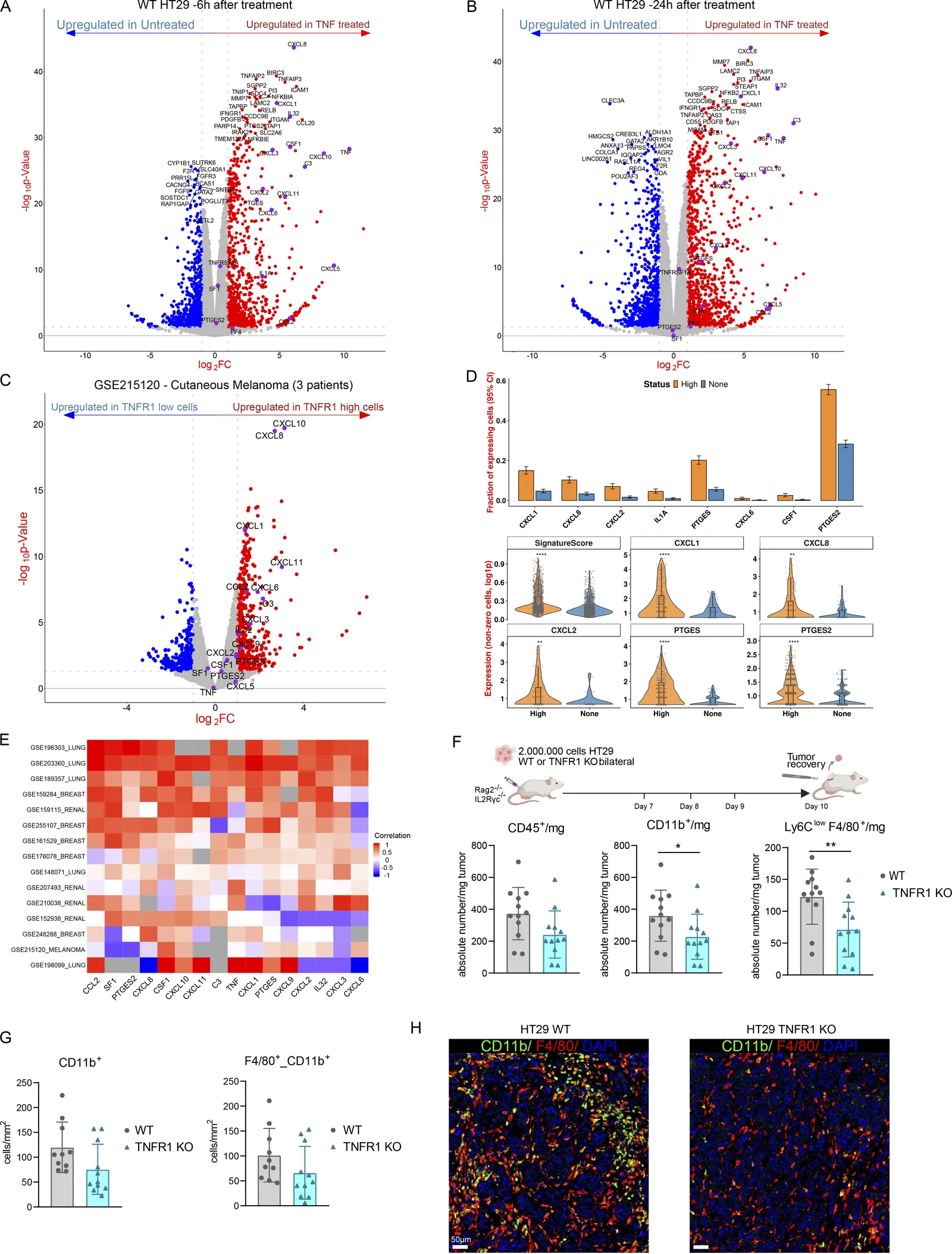
**TNFR1 on human tumor cells mediates pro-tumor inflammation. (A and B)** Volcano plots of bulk RNA-seq, as in Fig. 4 performed on HT29 tumor cells in culture, either exposed or not to recombinant human TNFα, highlighting pro-tumor inflammatory mediators in purple (*n* = 3). **(C)** Volcano plot representing a comparison of expression of mRNA in the malignant melanoma cells (scRNA-seq) from three cases of primary resections of skin melanoma that were in silico sorted into TNFR1^high^ and TNFR1^negative/low^. Genes of the proinflammatory signature identified in A and B are highlighted in purple. **(D)** Bar plot (upper panel) and violin plots (lower panel) representing the gene expression distribution for the main genes of the proinflammatory signature across TNFR1^high^ and TNFR1^negative/low^ cells in two resected NSCLC cases, again sorted in silico. **(E)** Heatmap representing the coefficients of linear correlation (Pearson’s) between TNFR1 transcripts in malignant cells in the indicated datasets (representing several solid malignant diseases) and the expression of the indicated proinflammatory genes. **(F)** Experiments in Rag2^−/−^IL-2Rγ^−/−^ immunodeficient mice subcutaneously xenografted with TNFR1KO or WT HT29 cells whose tumors were removed on day 10 as indicated in the scheme to generate cell suspensions which were studied for the intratumoral content of mouse myeloid cells. Each dot represents data from an individual tumor (*n* = 12). **(G)** Multiplex tissue immunofluorescence analyses of excised samples as in F (*n* = 10–11). **(H)** Representative microscopy images (Scale bar: 50 µm). A *t* test statistical comparisons were shown (F and G). P < 0.05 (*), P < 0.01 (**), and P < 0.0001 (****).

We next sought to investigate whether these mechanisms could be operating in human cancer cells. We first performed bulk RNA-seq experiments on HT29 human colon cancer cells upon exposure to human rTNFα. Again, 6 and 24 h results indicate the induction of multiple secondary inflammatory mediators in these cultures (Fig. 5, A and B).

To ascertain if the levels of TNFR1 on human malignant cells were associated with their expression of secondary inflammatory factor transcripts, we made use of publicly accessible scRNA-seq datasets with sufficient malignant cell numbers and sequencing depth. In a discovery dataset encompassing three primary skin melanomas (Fig. 5 C), we selected melanoma cells using exclusively expressed mRNA markers. Among them, we in silico separated those expressing TNFR1 above a threshold based on the local minimum in the expression density of TNFR1 to be analyzed in comparison with those melanoma cells expressing none or weak TNFR1 mRNA. As observed in Fig. 5 C, transcripts encoding secondary inflammatory mediators (depicted in purple) were selectively more expressed in the TNFR1^high^ melanoma cell populations, chiefly including IL8 (CXCL8). IL-8 (CXCL-8) is a prominent target and a chemokine known to interfere with cancer immunotherapies in patients (Alfaro et al., 2017; Schalper et al., 2020; Teijeira et al., 2020; Bertino et al., 2021).

Using a similar strategy on scRNA-seq results, we compared TNFR1^high^ versus TNFR1^negative/low^ in lung carcinoma cells from two resected NSCLC patients. The bar graphs and violin plots in Fig. 5 D confirmed the overexpression of the key secondary proinflammatory factors in TNFR1^high^ epithelial malignant cells identified with a set of mRNA markers.

Moreover, we analyzed a large series of scRNA-seq datasets across solid malignant diseases to define the correlation between TNFR1 transcript expression levels in malignant cells and those of the main secondary proinflammatory factors identified in Fig. 5, A–C. The heatmap in Fig. 5 E shows a clear association tendency (Pearson's linear correlation coefficient between the level of TNFR1 expression in malignant cells and their expression of the secondary proinflammatory genes). These associations are consistent with a role for TNFR1 on malignant cells themselves to orchestrate a proinflammatory milieu of secondary factors. Of note, there are also technical limitations related to the number of malignant cells retrievable for in silico scRNA-seq analyses and difficulties to accurately determine TNFR1-negative cells due to the intrinsic sensitivity of scRNA-seq and spatial profiling methods.

**Figure S3.**
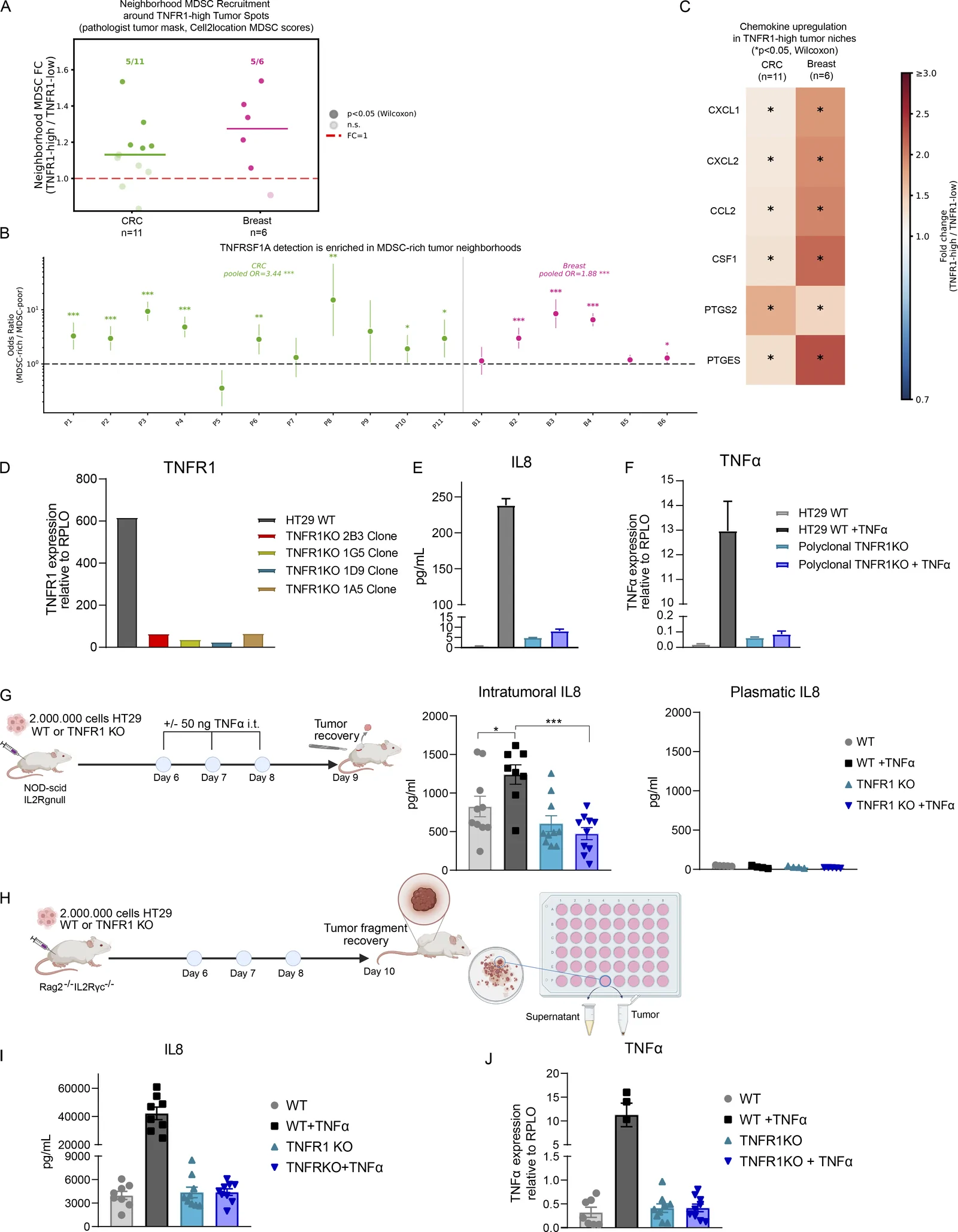
**Human evidence for a proinflammatory immunosuppressive role of TNFR1 expressed by malignant cells. (A)** Bioinformatic analyses of digital spatial transcriptomics (Visium) of tumor niches in a series of colon and breast cancer analyses showing that tumor TNFR1-enriched areas express more prominently transcripts associated with MDSCs. **(B)** Patient-by-patient sample analyses show that MDSC-enriched tumor niches show more TNFR1 expression in malignant cells. A one-sided Mann–Whitney U test was performed. **(C)** Correlations of the transcriptional expression of the indicated inflammatory mediators with respect to TNFR1 expression in the tumor niches (Wilcoxon test). **(D)** mRNA expression of TNFR1 quantified by quantitative RT-PCR in WT HT29 cells and four CRISPR/Cas9 TNFR1KO clones. **(E)** IL-8 concentration levels after TNFα stimulation in WT and TNFR1KO HT29 cells, showing that the induction of IL-8 is lost in the TNFR1KO variants (*n* = 6–12). **(F)** TNFα gene expression levels relative to RPLO mRNA levels quantified by quantitative RT-PCR in WT and TNFR1KO cultured variants, showing that the induction of TNFα transcripts by human rTNFα is lost in the TNFR1KO variants (*n* = 6). **(G)** NSG immunodeficient mice were subcutaneously xenografted with either HT29 WT or TNFR1KO variants, and tumors were injected with or without rTNFα, as indicated. 9 days after tumor-cell inoculation, tumors were excised, and IL-8 concentrations (mean + SEM and *t* test for statistical comparisons) were assessed in the tumor tissue homogenate fluid (left) and in plasma (right) (*n* = 10). **(H)** Schematic representation of experiments in which immunodeficient Rag2^−/−^IL-2Rγ^−/−^mice were xenografted with HT29 tumors, either HT29 WT or HT29 TNFR1KO. 10 days after engraftment, tumors were excised, and small tumor fragments (approx. 1 mm × 1 mm) were set in culture as shown in the scheme, in the presence of either medium alone or rhTNFα. **(I and J)** IL-8 concentrations were monitored in the culture supernatants (I), and TNFα transcripts were assessed by quantitative RT-PCR in the cell pellets (J) (*n* = 8–9). P < 0.05 (*), P < 0.01 (**), and P < 0.001 (***).

Another prediction, given TNFR1 heterogeneity in expression level, is that TNFR1^high^ malignant cells should be more profusely surrounded by myeloid cells. To address this point, we used publicly available digital spatial profiling datasets (Visium) from colon cancer and breast cancer, comparing niches with high TNFR1 in malignant cells to those with lower expression of the transcript. Interestingly, high TNFR1 content was relatively enriched in MDSC gene signatures (Fig. S3 A). Reciprocally, those tumor neighborhoods with higher content of MDSC were associated with higher TNFR1 expression in malignant cells across patients (Fig. S3 B). Furthermore, a positive association was found between TNFR1 expression in the tumor niches and co-expression of inflammatory mediators (Fig. S3 C).

Although these associations were substantiated in an important number of human cancer cases, the correlations were not perfect and may indicate patient heterogeneity regarding other primary proinflammatory mediators alongside TNFα, as well as in the secondary mediators induced. It is therefore paramount to consider that TNFα might not be the only factor orchestrating the proinflammatory status of malignant cells.

### TNFR1 on human cancer cells is functional to induce secondary inflammatory mediators and to chemoattract MDSC

Next, we knocked out TNFR1 using three commercial CRISPR/Cas9 guides in the human HT29 colon cancer cells. In this way, we generated TNFR1-negative clonal variants (Fig. S3 D) that lost the prominent induction of CXCL8 (IL-8) by rTNFα (Fig. S3 E) that we had previously reported (Olivera et al., 2022). Interestingly, TNFα gene expression was also induced by TNFα incubation of these cultured HT29 cells, and its induction was lost in the TNFR1KO variants (Fig. S3 F).

Moreover, when these tumor cells were xenografted in NSG mice and intratumorally injected with human recombinant TNFα, increased concentrations of IL-8 (CXCL-8) were detected in the interstitial fluid of excised tumors, while IL-8 was not increased in circulation (Fig. S3 G). This function was lost when HT29 TNFR1KO cells were similarly xenoengrafted (Fig. S3 G). However, our analysis of transcriptomic datasets from human tumors has limitations and remains correlative without offering a mechanistic link between cancer cell-intrinsic TNFR1 signalling and the accumulation and/or functional programming of the myeloid cells in human cancer tissue.

To assess the functionality of TNFR1 on human tumor cells, we took advantage of the fact that human CXCL8 (IL-8) chemoattracts mouse myeloid cells (Teijeira et al., 2021). Hence, we xenografted HT29 TNFR1KO and WT variants into Rag2^−/−^IL2Rγ^−/−^ mice and excised the tumors 10 days later. Tumor fragments (∼1 × 1 mm in size) were set in 72 h cultures with or without hrTNFα (Fig. S3 H). In these cultures, IL-8 soluble protein and TNFα gene expression (Fig. S3 J) were increased by rTNFα, but only when TNFR1 remained expressed on the tumor cells.

More importantly, we observed that TNFR1-expressing xenografted tumors contained more mouse myeloid cells as assessed by multicolor flow cytometry on cell suspensions (Fig. 5 F) and confirmed by multiplex tissue immunofluorescence (Fig. 5 G). Accordingly, our human results collectively recapitulate mouse observations regarding the consequences of TNFR1 expression on tumor cells, at least for the most part.

All considered, we provide novel solid evidence for a tumor-intrinsic TNFα→TNFR1 stimulation of immunosuppressive inflammatory mechanisms that critically allow for immune escape, shaping the tumor microenvironment. Thus, our results keep advocating for clinical trials testing TNFα neutralization in combined regimens with standard or experimental immunotherapies (Perez-Ruiz et al., 2019).

## Materials and methods

### Cell lines and CRISPR/Cas9 engineering

MC38 cell line (RRID:CVCL_B288) was provided by Dr. Karl E. Hellström (University of Washington, Seattle, WA, USA) in 1998. E0771 cell line (RRID:CVCL_GR23) was acquired from ATCC in 2017. B16-OVA cell line (RRID:CVCL_WM78) was a kind gift of Dr. Lieping Chen (Yale University, New Haven, CT, USA) in 2001. CT26 cell line (RRID:CVCL_7254) was kindly provided by Mario Colombo (IRCCS Istituto Nazionale dei Tumori, Milano, Lombardia, Italy). 4T1 breast carcinoma cells (RRID:CVCL_0125) of BALB/c origin were originally provided by Dr. Claude Leclerc (Institute Pasteur, Paris, France), and verified in the master cell bank at the Institute Pasteur. Panc02 cell line (RRID:CVCL_D627) was isolated from Panc02 tumor tissue obtained from the National Cancer Institute, DCTDC Tumor Repository (Frederick, MD, USA) in 2003 by Guillermo Mazzolini (Mazzolini et al., 2003). LLC cell line (RRID:CVCL_4358) was kindly provided by Karmele Valencia (Centro de Investigación Médica Aplicada [CIMA]; University of Navarra, Pamplona, Spain). B16F10 (RRID: CVCL_0159) cells were purchased from the ATCC in June 2006. HT29 human cell line (RRID: CVCL_0323) was acquired from ATCC in 2011. Tumor cell lines were cultured in RPMI 1640 medium (Gibco; RRID:SCR_021147) supplemented with 10% FBS (Sigma-Aldrich; RRID:SCR_021152), 100 U/ml penicillin/100 µg/ml streptomycin (Gibco; RRID:SCR_021155), mouse cell lines were supplemented with 5 × 10^−5^ mol/l 2-mercaptoethanol (Gibco; RRID:SCR_021156), and B16-OVA cell line was also supplemented with 400 μg/ml of geneticin (Gibco; RRID:SCR_021157). All tumor cell lines were maintained in a humidified incubator at 5% CO_2_ and 37°C for at least 7 days before being inoculated into mice. Mycoplasma contamination tests were routinely performed on all cell lines using the MycoAlert Mycoplasma Detection Kit (Lonza; RRID:SCR_025549).

To generate murine TNFR1KO cell variants, each cell line was seeded at high cell density, 1 day prior to transfection with a customized vector from GenScript encoding a gRNA, Cas9, and GFP, using Lipofectamine 2000 (Thermo Fisher Scientific; RRID:SCR_025551). gRNA was designed using several platforms (Benchling, Breaking-Cas, CHOP-CHOP, and Cas-OFFinder), which predict the probability of on- and off-target gene editing. gRNA with the highest score across multiple platforms was selected. gRNA sequence is provided in Fig. S1. The gene-edited cells were sorted out twice using a MoFlo Astrios EQ sorter (Beckman Coulter; RRID:SCR_018893): first by GFP expression, and then by the absence of TNFR1 expression stained with anti-CD120a (113003; BioLegend; RRID:AB_313532) and single–well-seeded to obtain several monoclonal TNFR1KO cell lines.

CRISPR/Cas9-mediated TNFR1 gene editing was also validated in all cell lines by Sanger sequencing analysis. To prepare a cell sample for Sanger sequencing, genomic DNA was first extracted (69504; Qiagen; RRID:SCR_025552) and purified (740609; NucleoSpin; RRID:SCR_025553), following the manufacturer’s instructions. A specific region of interest was amplified by PCR using gene-specific primers (5′-ATT​TTG​CTG​CCC​CAC​TCC​CTG​C-3′; 5′-TAC​CGC​CAC​ACT​ACG​AGC​AGA-3′). The purified PCR product is then quantified and submitted for Sanger sequencing in the genomic facility at CIMA. The resulting chromatograms were analyzed using the TIDE (Tracking of Indels by Decomposition), a web tool (https://tide.nki.nl/) to assess genome editing efficiency by quantifying the frequency and nature of indels introduced at the target site.

The HT29 TNFR1KO cell variants were established using the TNF-R1 CRISPR/Cas9 KO plasmid (h2) from Santa Cruz (sc-400206-KO-2 RRID:SCR_025554). The gene-edited cells were sorted out by GFP using a MoFlo Astrios EQ sorter (Beckman Coulter; RRID:SCR_018893) to obtain several monoclonal TNFR1KO cell lines, which were validated by real-time quantitative PCR (5′-TGG​TGG​GAT​ATA​CCC​CTC​AG-3′; 5′-GCA​CTT​GGT​ACA​GCA​AAT​CGA​AT-3′) and by functional analysis. Each cell line was seeded at high cell density, and 1,000 U/ml of rTNFα (300-01A; PeproTech; RRID:SCR_025539) was added. After 72 h, supernatants were collected, and an IL8 ELISA (555244; BD; RRID:SCR_025547) assay was performed. Those clonal variants with less TNFR1 expression by RT-PCR and lower levels of IL8 secretion after TNFα exposure were chosen and mixed for a polyclonal culture.

### Lentiviral transfection of TNFR1 and cell sorting

EO771 cells were transduced with pLV[Exp]-Puro-EF1A>mTnfrsf1a lentiviral vectors carrying mouse TNFR1 (purchased from Vector Builder). 5 × 10^5^ EO771 cells seeded in 6-well plates were transduced with viral vectors (MOI of 2.5) for 12 h at 37°C along with 5 μg/ml polybrene. Cells were selected in RPMI with 5% FBS and 2.5 μg/ml puromycin for 5 days. After that, cells were stained with anti-CD120a (113003; BioLegend; RRID:AB_313532) and FACS sorted for regained TNFR1 expression in a MoFlo Astrios EQ sorter (Beckman Coulter; RRID:SCR_018893).

### Incucyte proliferation assays

MC38 and EO771 WT and TNFR1KO cells were seeded at a density of 3 × 10^5^ cells/well in a 96-well flat-bottom plate. Plates were placed into the IncuCyte SX5 Live-Cell Analysis System (RRID:SCR_026298) housed within a standard tissue culture incubator (37°C, 5% CO_2_). Phase-contrast images were captured every 6 h using a 20× objective lens. Cell proliferation was quantified by analyzing cellular confluence (%) over time using the IncuCyte Base Analysis Software.

### Mouse strains and in vivo experiments

All mouse experiments were approved by the ethics committee for animal experimentation of the regional government of Navarra under Spanish regulations (078/21, 077/21, 079/20). 5- to 6-wk-old C57BL/6 mice were purchased from Envigo. Mice were maintained in the animal facility at CIMA under standard conditions, in accordance with institutional guidelines. Rag1^−/−^, Rag2^−/−^IL2Rγc^−/−^, BATF3^−/−^ (Hildner et al., 2008), and NSG mice were bred in our facilities (CIMA).

To analyze the tumor engraftment capability following TNFR1 silencing, C57BL/6, Rag1^−/−^ or Batf3^−/−^ mice were subcutaneously injected on day 0 with 0.5 × 10^6^ of WT or TNFR1KO tumor cell lines (MC38, E0771, B16-OVA, CT26, Panc02, LLC, 4T1, or B16F10) suspended in 50 μl of PBS. In some experiments, tumor engraftment was evaluated using concomitant subcutaneous tumors (TNFR1KO tumor cells in one flank and WT tumor cells in the opposite flank) or mixtures of WT and TNFR1KO cells at a 1:5 or 1:1 ratio. For rechallenge experiments, those mice that rejected TNFR1KO tumors and naive mice were subcutaneously injected with the corresponding WT cell line and with B16OVA in the contralateral flank.

To study the role of each immune cell population in the rejection of tumor engraftment in TNFR1KO variants, depletions of different immune cell subsets were performed in MC38 or EO771 tumor-bearing mice by intraperitoneal injection of 100 µg of anti-CD8_β_ (BE0223; BioXcell; RRID:AB_2687706), anti-CD4 (BE0003; BioXcell; RRID:AB_1107636), or Rat IgG (BioXcell; BE0094; RRID:AB_1107795) isotype control antibodies, starting the prior day to cell inoculation and given twice a week during the experiments.

For HCC experiments, hydrodynamic tail-vein injection was performed as previously described (Ochoa et al., 2023; Ruiz de Galarreta et al., 2019). pT4-EGFP was cloned using the vector pT4/HB (108352; Addgene), and the EGFP insert was included in the HindIII/XbaI site, and pT3-c-myc-luc (129775; Addgene), pCMV-SB13, and pgRNAp53-Cas9 (px330-sg-p53) plasmids were kindly provided by Dr. Lujambio (Icahn School of Medicine at Mount Sinai, New York, NY, USA). A volume of 2 ml of saline dilution containing the appropriate plasmid concentration was hydrodynamically injected through the tail vein. Each mouse received 36 μg of pT3-c-myc-luc, 7.5 μg of pCMV-SB13, 30 μg of pgRNAp53-Cas9, 10 µg of pT4-EGFP, and 40 µg of TNFR1-CRISPR/Cas9 plasmid or the corresponding empty vector as a control. Luciferase was quantified by bioluminescence at different time points in an IVIS Spectrum system (Perkin Elmer; RRID:SCR_018621). Light intensity was quantified using photons/sec/cm^2^/sr; animals with <1 × 10^8^ photons/sec/cm^2^/sr were considered tumor free. The color-scale photograph and data images were superimposed using Living Image version 4.4 software (Perkin Elmer; RRID:SCR_014247).

### Flow cytometry on tumor-derived cell suspensions

To analyze tumoral immune cell infiltration, C57BL/6 mice were injected with 0.5 × 10^6^ of either WT or TNFR1KO variants of the MC38 or E0771 cell lines. Mice received three doses of 50 ng of TNFα intratumorally at indicated time points in the experimental workflow (Fig. 4, A and B). 24 h after the last dose of TNFα, tumor samples were collected and assessed individually by flow cytometry. Prior to flow cytometry staining, all tumor samples were incubated in collagenase/DNase I for 15 min at 37°C. Then, all the specimens were mechanically disaggregated and filtered through a 70-μm cell strainer (Thermo Fisher Scientific). Each specimen was surface-stained with the following fluorochrome-labelled antibodies: FcR-Block (101320; anti-CD16/32 BioLegend; RRID:AB_1574975), anti-CD45.2-FITC (109806; BioLegend; RRID:AB_313443), anti-CD4-BUV496 (612952; BD Bioscience; RRID:AB_2813886), anti-CD8-AF700 (100730; BioLegend; RRID:AB_493703), anti-CD11b-BUV395 (563553; BD Bioscience; RRID:AB_2738276), anti-Ly6C-AF467 (128010; BioLegend; RRID:AB_1236550), anti-Ly6G-BV510 (127633; BioLegend; RRID:AB_2562937), anti-F4/80PECy7 (123114; BioLengend; RRID:AB_893478), and anti-CD3-PerCPeF710 (46-0032-82; eBioscience; RRID:AB_1834427).

To determine tumoral immune cell infiltration in HT29 tumor-bearing mice, Rag2^−/−^ IL2Rγc^−/−^ mice were injected subcutaneously with 2 × 10^6^ cells of WT or TNFR1KO HT29 cell lines. On day 10, tumor-derived cell suspensions were analyzed. Each tumor sample was surface-stained using the following mixture of fluorochrome-labelled antibodies: anti-CD45.2 FITC (109806; BioLegend; RRID:AB_313443), anti-Ly6C-AF647 (128010; BioLegend; RRID:AB_1236550), anti-Ly6G-BV510 (127633; BioLegend; RRID:AB_2562937), anti-CD11b-BV650 (101259; BioLegend; RRID:AB_2566568), and anti-F4/80-BV421 (123132; BioLegend; RRID:AB_2563102).

In those experiments in which we analyzed the levels of TNFα in tumors from MC38, EO771 and B16F10, we used the tumor dissociation kit (130-096-730; Miltenyi Biotec; RRID:SCR_020285) following the manufacturer’s instructions and using the gentleMACS Octo Dissociator (Miltenyi Biotec). Each sample was surface-stained with the following fluorochrome-labelled antibodies: anti-CD45.2 (109806; FITC BioLegend; RRID:AB_313443), anti-TNFα BV421 (506327; BioLegend; RRID:AB_10965355), Rat IgG1 (401921; BioLegend; RRID:AB_2562601). All the process was done in the presence of GolgiStop Protein Transport Inhibitor (554724; BD Biosciences; RRID:AB_2869012).

For the in vivo assessment of TNFR1 WT:KO mixture experiments, we used the tumor dissociation kit (130-096-730; Miltenyi Biotec; RRID:SCR_020285) following the manufacturer’s instructions and using the gentleMACS Octo Dissociator (Miltenyi Biotec). The samples were stained with anti-CD45 BUV496 (569670; BD Horizon, RRID:AB_3685217), anti-CD11b FITC (101206; BioLegend, RRID:AB_312789), anti-TNFR1 PE (113003; BioLegend; RRID:AB_313532), anti-CD31 BV421 (562939; BD Horizon; RRID:AB_2665476), anti-CD90.2 BV605 (105343; BioLegend; RRID:AB_2632889), and Armenian Hamster IgG (400908; BioLegend; RRID:AB_326593).

The Zombie NIR Fixable viability kit (423106; BioLegend; RRID:SCR_023773) or 7AAD Viability Kit (420404; BioLegend; RRID:SCR_023774) was used as a live/dead cell marker.

All flow cytometry stainings were analyzed using a CytoFLEX (Beckman Coulter; RRID:SCR_019690) cytometer. Results were normalized to tumor weight and expressed as cells per mg of tumor tissue.

### Multiplex tissue immunofluorescence staining and analysis

Immunofluorescence staining and analysis were performed as previously described on a Bond RX autostainer (Abengozar-Muela et al., 2020; López-Janeiro et al., 2022). 4-μm-thick FFPE tissue sections were deparaffinized (Bond Dewax, Leica Biosystems; RRID:SCR_025531) and rehydrated per standard protocols. Antigen retrieval was performed with Bond Epitope Retrieval Solution 2 (ER2, AR9640; Leica Biosystems; RRID:SCR_025532), followed by primary antibody incubation (Akoya antibody diluent/block; RRID:AB_3094498) or two sequential cycles of staining, with each cycle including a 30-min combined block and primary antibody incubation (Akoya antibody diluent/block; RRID:AB_3094498), followed by a secondary HRP-conjugated polymer. Signal amplification was achieved with TSA-Opal fluorophores. The primary antibodies and corresponding fluorophores are anti-CD11b (rabbit polyclonal, dilution: 1:2,000, NB110-89474; Novus Biologicals; RRID:AB_1216361) in Opal 520; anti-F4/80 (rabbit monoclonal, clone EPR26545-166, dilution: 1:1,000; ab300421; Abcam; RRID:AB_2936298) in Opal 690. Nuclei were counterstained with Spectral DAPI (FP1490; Akoya Biosciences, RRID:SCR_025533) and the stained tissues were mounted with ProLong Diamond Antifade mounting medium (Thermo Fisher Scientific; RRID:SCR_015961). Stained slides were scanned using the PhenoImager HT Automated Quantitative Pathology Imaging System (Akoya Biosciences; RRID:SCR_023772). After image acquisition, unmixing of the spectral libraries was performed with inForm software (Akoya Biosciences). Unmixed images were then imported into the open-source digital pathology software QuPath version 0.4.4 for cell segmentation and cell phenotyping. Marker expression was used to identify CD11b^+^ and F4/80 cells. Cell densities of each cell population were quantified.

### Quantitative RT-PCRs and ELISAs

Total RNA was extracted from murine and human tumor cell lines with the Maxwell RSC simply RNA extraction kit (AS1340; Promega; RRID:SCR_025534) according to the manufacturer’s instructions and subsequently retrotranscribed into cDNA using the M-MLV enzyme kit (28025013; Invitrogen) RRID:SCR_025535. Real-time PCR reactions were performed in the Bio-Rad CFX qPCR system (RRID:SCR_018064) with customized primers for mTNFα (FW 5′-CTA​TGT​CTC​AGC​CTC​TTC​TC-3′ and RV 5′-CAT​TTG​GGA​ACT​TCT​CAT​CC-3′) and human TNFα (FW 5′-GAC​ACC​ATG​AGC​ACT​GAA​AGC-3′ and RV 5′-AGC​TTG​AGG​GTT​TGC​TAC​AAC-3′).

For assessing immune cytokine production in response to TNFα in mouse tumor cell lines (EO771 and MC38), 10^5^ cells were seeded in a 48-well plate. 3 days after stimulation with TNFα (1,000 U/ml; PeproTech; 300-01A; RRID:SCR_025539), culture supernatants were collected to measure the concentrations of CXCL1 and CXCL2 by ELISA assays (SMKC00B; R&D Systems; RRID:SCR_025540 and MM200; R&D Systems; RRID:SCR_025541, respectively). CCL2 levels were quantified using the CCL2/JE/MCP-1 ELISA kit (MJE00B; R&D Systems; RRID:SCR_025542), M-CSF using the M-CSF ELISA kit (MMC00B; R&D; RRID:SCR_025543), PGE_2_ using the Prostaglandin E_2_ ELISA kit (ab287802; Abcam; RRID:SCR_025544), and prostaglandin E synthase using the MPGES-1 ELISA kit (A75765; Antibodies; RRID:SCR_025545).

To investigate cytokine production in response to TNFα in HT29 cell line, Rag2^−/−^ IL2Rγc^−/−^ or NSG mice were injected subcutaneously with 2 × 10^6^ of WT or TNFR1KO HT29 cells. On day 10, plasma or tumor samples were collected. Tumors were manually cut into ∼1 mm^3^ pieces on ice. Three tumor fragments were seeded in a 96× flat-bottom plate and cultured in 250 μl of culture medium in the presence of rTNFα (1,000 U/ml, 300-01A; PeproTech; RRID:SCR_025539). 72 h after, supernatants were collected to assess IL-8 concentrations using a human IL-8 ELISA kit (555244; BD; RRID:SCR_025547).

### Tumor cell lines bulk RNA-seq sequencing and analysis

Bulk RNA-seq transcriptomic analysis of EO771 and HT29 tumor cell lines was collected at 6 and 24 h after treatment with 1000 U/ml of TNFα (300-01A; PeproTech; RRID:SCR_025539). RNA was extracted using the RNeasy Mini Kit (74104; Qiagen; RRID:SCR_025548) and sent for RNA-seq sequencing analysis to Macrogen. Data generated from bulk RNA-seq experiments were deposited in the Gene Expression Omnibus (GEO) database and can be obtained from the following accession codes: GSE309307 for mouse cell line data, and GSE309029 for human cell line data. Raw RNA-seq sequencing data were processed as follows. Quality control (QC) and trimming of adapter sequences were done with fastQC (version 0.11.9) and Trimmomatic (version 0.39), respectively. Alignment to the reference genome hg38 GENCODE Reference 39 (in human) and mm10 GENCODE Reference M27 (in mouse) was performed with STAR (version 2.7.11b). Final quantification and raw counts matrix were obtained using featureCounts (version 2.0.6). Data were then analyzed using R (version 4.5.1) through RStudio. Data management was performed using the SummarizedExperiment package (version 1.38.1), in conjunction with the edgeR (version 4.6.3) pipeline for differential gene expression analysis. Gene Set Enrichment Analysis (GSEA) was performed using clusterProfiler (version 4.16.0). Gene sets used for GSEA analysis were obtained from MSigDB database through the msigdbr (version 25.1.0) R package, and all GO pathways and Hallmark gene sets were considered. All plotting was performed using ggplot2 (version 3.5.2) and enrichplot (version 1.28.2). All these packages, among other packages implied in dimensionality reduction and other statistical parameters such as scater (version 1.36.0) are part of the Bioconductor project (version 3.21). For these techniques, multiple testing correction was performed using the Benjamini–Hochberg false discovery rate method. In many figures, a gene signature composed of secondary inflammatory mediators was curated and highlighted. This signature is composed of the genes: TNFRSF1A, CCL2, IL1A, C3, CXCL1, CXCL2, CXCL3, PF4, CXCL5, CXCL6, CXCL9, CXCL10, CXCL11, SF1, CSF1, TNF, PTGES, PTGES2, IL32, and CXCL8.

### CD11b+ myeloid cell sorting and bulk RNA-seq

EO771 WT or TNFR1KO tumors were excided at day 8 after inoculation and processed with the tumor dissociation kit (130-096-730, Miltenyi Biotec; RRID:SCR_020285) following the manufacturer’s instructions and using the gentleMACS Octo Dissociator (Miltenyi Biotec; RRID:SCR_020284). Cell suspensions were stained with anti-CD45.2 FITC (109806; BioLegend, RRID:AB_313443) and anti-CD11b PE (101208; BioLegend, RRID:AB_312791) for FACS-sorting of CD11b^+^ cells in a MoFlo Astrios EQ sorter (Beckman Coulter; RRID:SCR_018893). The sorted cells were sent for RNA-seq sequencing analysis to Dreamgenics (Spain). Data generated from bulk RNA-seq were deposited in the GEO database and can be obtained from accession no. GSE334550.

Tumor suspension raw data in fastq format were processed using the nf-core/rnaseq pipeline (version 3.22.2), executed via Nextflow (version 25.10.2). QC was performed with FastQC (version 0.12.1), trimming with Trim Galore (version 0.6.10) and Cutadapt (version 4.9). Trimmed reads were pseudo-aligned and quantified against the mouse reference transcriptome (ENSEMBL GRCm39 release 113) using Salmon (version 1.10.3) in quasi-mapping mode with a k-mer size of 20. Transcript-level abundance estimates were aggregated to gene-level counts using tximeta (version 1.20.1).

Data were then analyzed using R (version 4.4.1) through RStudio, imported as a SummarizedExperiment (version 1.38.1) object. Metadata was generated for further analysis, classifying samples into two condition groups: TNFR1KO tumors and TNFR1WT tumors. Gene identifiers were mapped from Ensembl IDs to Gene Symbols using the org.Mm.eg.db (version 3.21.0) package. Data were coerced into a SingleCellExperiment (version 1.30.1) object. Log-normalization of counts and principal component analysis were done using scater (version 1.36.0). We checked for potential B cell contamination, finding very small amounts of their related cells (<0.1% cumulative sample proportion), therefore excluding immunoglobulin-related genes and other non-annotated transcripts and pseudogenes from the formal analysis.

Differential gene expression analysis was performed as per the edgeR (version 4.6.3) pipeline. GSEA was performed using clusterProfiler (version 4.16.0), employing gene sets obtained from MSigDB through the msigdbr package (version 26.1.0). Reactome and Hallmark gene sets were used in GSEA.

To quantify phenotypic activation profiles on a per-sample basis, we performed Gene Set Variation Analysis on the log-transformed counts per million (CPM) expression matrix. It was performed through the GSVA R package, using a Gaussian kernel cumulative density function. We performed such analysis with the goal of establishing general myeloid axes, mapping standard functional programs with dedicated gene sets such as the T cell suppression signature (*Ido1*, *Cd274*, *Lgals9*, *Tgfb1*, *Il10*, *Entpd1*, *Arg1*, *Vegfa*, *Il4ra*, and *Pges*).

### Biometric analyses of spatial transcriptomics

Spatial transcriptomics data were obtained from two independent cohorts (Zenodo: https://doi.org/10.5281/zenodo.7551712, GEO: GSE176078), as reflected in the data availability section. The colorectal cancer (CRC) dataset consists of 11 tumor sections, while the breast cancer dataset comprises six samples (four triple-negative breast cancer and two ER^+^).

Analysis was restricted to tumor-bearing sections, and spot-level pathologist annotations served as ground truth for tumor compartment definition in both cohorts.

Visium spatial transcriptomics captures bulk gene expression from spots containing multiple cell types (∼10–20 cells per spot). To estimate the abundance of specific cell types within each spot, spatial deconvolution was performed using Cell2location (version 0.1) to estimate local cell type abundances based on cohort-specific reference atlases. For the CRC cohort, pre-computed abundance profiles were obtained from Valdeolivas et al. (2024), which utilized the colorectal scRNA-seq atlas from Lee et al. (2020). For the breast cancer cohort, raw Visium matrices were deconvolved against the paired scRNA-seq atlas from the same study (Wu et al., 2021; GSE176078; celltype_minor annotation). The breast cancer deconvoluted model (RegressionModel) was trained on the reference atlas (max_epochs = 250, GPU acceleration, batch_key = orig.ident), and posterior cell type signatures were exported using 1,000 posterior samples. Spatial deconvolution was executed per sample with the following parameters: N_cells_per_location = 8, detection_alpha = 20, max_epochs = 20,000, and batch_size = none.

Candidate MDSC proxies were identified by scoring myeloid subsets in each reference atlas against a composite transcriptional signature (S100A8, S100A9, CD14, ITGAM, and FCGR3A). In the CRC cohort, the SPP1+A macrophage subset was selected based on its established immunosuppressive function and tumor-associated phenotype in colorectal malignancy. In the breast cancer cohort, the monocyte subset exhibited the highest composite score, further supported by expression of S100A12, and was therefore used as the MDSC proxy. Total tumor cell abundance was calculated as the sum of CMS1–CMS4 subtypes for CRC and luminal and basal epithelial subtypes for breast cancer. Tumor spots were defined based on morphological annotations provided by a board-certified pathologist.

To determine whether TNFR1-high tumor spots preferentially attract MDSC-like cells, we conducted a per-sample spatial neighborhood analysis. For each pathologist-annotated tumor spot, the six nearest spatial neighbors were identified using Euclidean distance on pixel coordinates (scikit-learn NearestNeighbors, k = 6), corresponding to the first ring of direct physical contact (∼100 µm inter-spot distance) in the Visium array geometry. The neighborhood MDSC score was defined as the mean MDSC abundance across these six neighboring spots. Within each sample, tumor spots were stratified for TNFRSF1A expression (normalized to CPM + log1p) into TNFR1-high (top 20th percentile, Q80) and TNFR1-low (bottom 20th percentile, Q20) groups. The fold-change in neighborhood MDSC scores between the two groups was computed as the ratio of their means.

To corroborate the neighborhood recruitment patterns through an independent statistical framework and account for TNFRSF1A transcript dropout, we asked whether tumor spots located adjacent to MDSC-rich neighborhoods show a higher probability of TNFRSF1A detection. For each pathologist-annotated tumor spot, the neighborhood MDSC score was computed identically to the analysis described above, and spots were stratified within each sample into MDSC-rich (top quartile, Q75) and MDSC-poor (bottom quartile, Q25) neighborhoods. A one-sided Fisher’s exact test was used to compare TNFRSF1A detection rates (raw count >0) between MDSC-rich and MDSC-poor groups. Odds ratios (ORs) with 95% confidence intervals were calculated using the Woolf method, and P values were corrected across samples using the Benjamini–Hochberg procedure. A pooled Fisher’s exact test combining all tumor spots per cancer type was used to derive a global OR estimate.

To assess whether TNFR1-high tumor spots upregulate myeloid-recruiting chemokines, a pooled analysis was performed across all pathologist-annotated tumor spots per cancer type. Within each sample, spots were stratified into TNFR1-high (Q75) and TNFR1-low (Q25) groups based on CPM + log1p-normalized TNFRSF1A expression. Expression fold-change (TNFR1-high/TNFR1-low) was evaluated for a panel of NF-κB target genes implicated in myeloid recruitment and immunosuppression: CXCL1, CXCL2, CCL2, CSF1, PTGS2, and PTGES. For each gene, statistical significance was determined using a one-sided Mann–Whitney U test (P < 0.05).

### scRNA-seq data analysis

Datasets used for scRNA-seq analyses are publicly available, with the following GEO accession numbers: GSE215120 (melanoma) and GSE198099 (lung) for specific profiles based on TNFR1 expression. Datasets considered for calculated correlations are GSE159284, GSE161529, GSE176078, GSE248288 (breast carcinomas); GSE148071, GSE164983, GSE189357, GSE196303, GSE203360 (lung carcinomas); GSE152938, GSE159115, GSE207493, GSE210038 (renal carcinomas), as described in the data availability statement, and processed as follows. First, cells were subjected to QC steps, filtering them based on having more than 200 expressed genes, a mitochondrial gene content lower than 20% of the total expression, and being classified as singlets using scDblFinder (version 1.22.0) to remove doublets. Then, genes expressed in <3 cells were removed from the data to improve computational efficiency of the pipeline. Data were checked for batch effects and corrected with Limma as needed.

All datasets were processed separately and checked for biological heterogeneity across patients. SingleR (version 2.10.0) was used to annotate cell types, and scATOMIC (version 2.0.3) was applied to the cells classified as “epithelial” (or melanocytes in melanoma datasets) to determine their malignant nature. Malignant cells were then selected and analyzed for their distribution of expression for the TNFR1 transcript. A local minimum in the bimodal distribution of expression was then calculated and used as a threshold to define TNFR1 status. Cells above the threshold were classified as “high,” those below as “low,” and those with no expression as “none,” High- and none-TNFR1–expressing malignant cells were then subjected to a pseudobulking algorithm based on their sample of origin. We only retained samples with a cell number higher than 70 to ensure a grade of robustness for the data for a pseudobulking approach.

The pseudobulks generated in the previous step were then compared in each dataset to make differential gene expression analysis. The gene signature of secondary inflammatory mediators, generated by analyzing bulk RNA-seq data, was used once more to contrast the results obtained in these experiments and correlate them with TNFR1 expression.

### Statistical analysis

Data were analyzed using GraphPad Prism 8.0. Flow cytometry data were processed using FlowJo version 10 or CytExpert software. Results are presented as means with either SEM or standard deviation (SD), as indicated. Tumor growth in vivo experiments are represented as tumor area (mm^2^) and shown either as individual data points per mouse or as group means ± SEM. All in vivo experiments were performed at least twice with a minimum of six mice per group, unless otherwise specified in the figure legends. For lentivirally transfected variants regaining TNFR1 expression, areas under the tumor growth curves were analyzed with unpaired *t* tests.

Statistical analysis of differences between independent groups was performed using one-way ANOVA, Mann–Whitney U test (*n* < 12), or a *t* test (*n* > 12). Tumor growth differences were assessed using two-way ANOVA. A P value <0.05 was considered statistically significant. Statistically significant differences are indicated by asterisks as follows: P < 0.05 (*), P <0.01 (**), P < 0.001 (***), and P < 0.0001 (****).

In hydrodynamic hepatocellular experiments, statistical analyses were performed using Fisher’s exact test between the control and CRISPR TNFR1 groups at each time point.

Statistical analysis of scRNA-seq data comparisons was made using Pearson correlations over logCPM-normalized expression data (pseudobulked), and in comparisons across distributions of expression, the Mann–Whitney U test was applied over log1p (natural logarithm of 1 + x) normalized data. Wilson’s 95% confidence interval was used for the bar plot. The same statistical significance criteria as in previous analyses were used.

Statistical differences between the TNFR1KO and TNFR1 WT signature scores and individual gene expression scores were evaluated using the nonparametric Mann–Whitney U test through the ggpubr package. For visualization purposes, ggplot2 (version 4.0.2) and enrichplot (version 1.28.4) were used. All these packages, among other packages used in the analysis process, are part of the Bioconductor project (version 3.21).

Statistical significance in MDSC neighborhoods was evaluated using a one-sided Mann–Whitney U test (alternative: TNFR1-high >TNFR1-low), excluding samples with fewer than five spots in either group. Significance was defined at P < 0.05. Results are presented as the fraction of significant samples per cancer type, with the median sample-level FC indicated by a horizontal bar.

Spatial and statistical analyses from spatial transcriptomic analyses were implemented in Python (version 3.10) using the following libraries: scanpy (version 1.9), cell2location (version 0.1), pandas, numpy, scipy, scikit-learn, statsmodels, matplotlib, and seaborn.

### Online supplemental material

Fig. S1 provides the methodology and the engraftment of TNFR1-silenced murine cell lines. Fig. S2 contains mechanistic experiments following TNFR1 loss and the consequences regarding TNFR1KO cells when co-engrafted with WT variants. Fig. S3 describes consequences of TNFR1 loss in human colon cancer HT29 cells and the relation of MDSC infiltrates in human tumors with regard to TNFR1 expression on malignant cells.

## Data Availability

Data generated from bulk RNA-seq experiments were deposited in the GEO database and can be obtained from accession no. GSE309307 for mouse cell line data and GSE309029 for human cell line data. Cd11b^+^ myeloid bulk RNA-seq data can be obtained from the accession no. GSE334550. Datasets used for scRNA-seq analyses are publicly available, with the GEO accession nos. GSE215120 (melanoma) and GSE198099 (lung) for specific profiles based on TNFR1 expression. Additional datasets considered for correlations calculated are: GSE159284, GSE161529, GSE176078, GSE248288 (breast carcinomas); GSE148071, GSE164983, GSE189357, GSE196303, GSE203360 (lung carcinomas); GSE152938, GSE159115, GSE207493, and GSE210038 (renal carcinomas). Datasets used for spatial transcriptomics come from two independent cohorts: the CRC dataset comes from a 10x Genomics Visium dataset from Valdeolivas et al. (2024) (Zenodo: https://doi.org/10.5281/zenodo.7551712). For breast cancer, we used the Visium dataset from Wu et al. (2021) (GEO: GSE176078). Raw data will be provided by the corresponding author upon reasonable request.
